# Comparative genomic and metabolic analysis of three *Lactobacillus paracasei* cheese isolates reveals considerable genomic differences in strains from the same niche

**DOI:** 10.1186/s12864-018-4586-0

**Published:** 2018-03-20

**Authors:** Ewelina Stefanovic, Olivia McAuliffe

**Affiliations:** Department of Food Biosciences, Teagasc Food Research Centre, Moorepark, Fermoy, Ireland

**Keywords:** *Lactobacillus paracasei*, comparative genomics, dairy

## Abstract

**Background:**

Strains of *Lactobacillus paracasei* are present in many diverse environments, including dairy and plant materials and the intestinal tracts of humans and animals. Their adaptation to various niches is correlated to intra-species diversity at the genomic and metabolic level. In this study, we compared the genome sequences of three *L. paracasei* strains isolated from mature Cheddar cheeses, two of which (DPC4206 and DPC4536) shared the same genomic fingerprint by PFGE, but demonstrated varying metabolic capabilities.

**Results:**

Genome sizes varied from 2.9 Mbp for DPC2071, to 3.09 Mbp for DPC4206 and 3.08 Mpb for DPC4536. The presence of plasmids was a distinguishing feature between the strains with strain DPC2071 possessing an unusually high number of plasmids (up to 11), while DPC4206 had one plasmid and DPC4536 harboured no plasmids. Each of the strains possessed specific genes not present in the other two analysed strains. The three strains differed in their abundance of sugar-converting genes, and in the types of sugars that could be used as energy sources. Genes involved in the metabolism of sugars not usually connected with the dairy niche, such as *myo*-inositol and pullulan were also detected, but strains did not utilise these sugars. The genetic content of the three strains differed in regard to specific genes for arginine and sulfur-containing amino acid metabolism and genes contributing to resistance to heavy metal ions. In addition, variability in the presence of phage remnants and phage protection systems was evident.

**Conclusions:**

The findings presented in this study confirm a considerable level of heterogeneity of *Lactobacillus paracasei* strains, even between strains isolated from the same niche.

## Background

The genus *Lactobacillus* consists of more than 200 species and subspecies [1] present in various environments such as plants, fermented food products (dairy, meat, wine), and both the human and animal gastrointestinal and reproductive tracts [2, 3]. One of the most studied groups of this genus is the *Lactobacillus casei* group, which includes the species *Lactobacillus casei, Lactobacillus paracasei* and *Lactobacillus rhamnosus*. Strains of this group show remarkable ecological adaptability and have been isolated from all the typical habitats of lactobacilli [4, 5]. Such a diverse range of sources facilitated a broad spectrum of applications of strains of this species in dairy production (adjunct cultures), and in health-related (probiotics, bacteriocins) and biotechnological fields. These characteristics and potential applications make the species of the *L. casei* group one of the best explored within the *Lactobacillus* genus.

To date, the genome sequences of 118 *L. casei* and *L. paracasei* strains are publicly available, 21 of which are complete genome sequences (http://www.ncbi.nlm.nih.gov, last accessed in January 2018). The comparative genomic analysis of *L. casei* and *L. paracasei* genomes has revealed that, as in other *Lactobacillales*, there is an evolutionary trend towards minimisation of genome size through the decay of genes coding for functions not required for strains inhabiting specific niches. This loss of redundant genes has been shown to be followed by the acquisition of genes by horizontal gene transfer (HGT) as a response to niche adaptation [6]. The rich pool of available genome sequences enables the definition of the gene sets that are common to all strains (core genome), the genes present in only some of the strains (dispensable genome), or genes that are unique for a single strain (unique genes). Insights into the common and unique genes enable correlation of gene variations among different strains to the presence or absence of phenotypic traits [7]. The pangenome (or supragenome) comprises the union of all genes present within a selected genome set (species, genera or higher taxonomic group) [8]. *L. casei* and *L. paracasei* pangenome studies have confirmed the wide range of ecological niches that can be inhabited by strains of the *L. casei* group [4, 7, 9], arising from the variability of genes supporting utilisation of numerous energy sources and other specific genes contributing to efficient survival in habitats with differing environmental conditions.

The dairy niche represents a nutritionally rich habitat, and niche specialisation in dairy strains has led to substantial gene decay, which has limited their survival in more nutritionally scarce environments [5]. As a consequence, genomes of dairy isolates possess a higher ratio of pseudogenes, compared to non-dairy isolates [10]. Conversely, genomes of dairy specialists are abundant in sugar transportation, proteolytic and amino acid transportation-encoding genes that enable uptake of nutrients present in the dairy environment [6]. However, the isolation source does not necessarily correspond to the original niche in which a strain has evolved, as strains can change their habitats due to their adaptability. This is evident from genome content, where often unusual genes that are not expected for strains from a specific niche are present, suggesting that a strain may have transferred from one niche to another [11].

The aim of this study was to compare the genomic and metabolic characteristics of three *L. paracasei* strains that were isolated from mature Cheddar cheeses. Previously, these strains were selected based on the activity of the key enzymes involved in flavour production and their volatile profiles in cheese model systems [12, 13]. Genomic fingerprinting established that two of the strains (DPC4206 and DPC4536) showed identical PFGE profiles, despite demonstrating considerable differences in selected enzyme activities, such as cell envelope proteinase, aminopeptidases, aminotransferase and glutamate dehydrogenase [12]. Similarly, these two strains exhibited distinct differences when examined for the production of volatile flavour compounds in two cheese model systems [13]. The third strain (DPC2071), which differed considerably in its PFGE profile, possessed high levels of activity of enzymes of the proteolytic system, especially cell envelope proteinase, and exhibited one of the most distinct volatile profiles in cheese model systems [12, 13]. It was proposed that elucidation and comparison of the genomes of these three strains would enhance our understanding of the genetic basis of their different phenotypic characteristics.

## Methods

### Bacterial strains for comparative analysis

The three *L. paracasei* strains examined in this study were isolated from the non-starter microbiota of Cheddar cheese, and deposited in the DPC Culture Collection. The genomes of all three strains are available from public databases (accession numbers: NCSN01000000, NCSO01000000 and NCSP01000000, for strains DPC2071, DPC4206 and DPC4536, respectively). Details of genome sequencing and assembly were previously reported [14]. Contig mapping was performed using Mauve, with the genomes of *L. paracasei* ATCC 334, *L. casei* BDII and *L. casei* 12A as references for strains DPC2071, DPC4206 and DPC4536, respectively [15].

### Identification of strain-specific genes in each of the input genomes

Whole genome comparisons were undertaken using BLAST Ring Image Generator [16], and progressiveMauve alignments [15], in order to identify unique genomic regions belonging to each of the strains.

Clustered regularly interspaced short palindromic repeat (CRISPR) regions in each genome were identified using an online tool CRISPRfinder [17]. Viable and cryptic prophages within each of the genomes were detected using PHASTER tool [18]. Contigs representing plasmid sequences were predicted based on the presence of typical plasmid-associated genes, such as replication and mobilisation genes, or based on similarity to sequences of previously published plasmids (www.ncbi.nlm.nih.gov).

### Plasmid profiles

Plasmid DNA was isolated using a method previously described [19]. Plasmid DNA was run on a 0.7 % (w/v) agarose gel, and visualised by staining with ethidium bromide.

### Modified media to assess carbohydrate fermentation

Modified MRS broth (MMRS) was made by the omission of beef extract and any other additional sugar source and was subsequently used as a medium to examine the growth of three strains in the presence of different carbohydrate substrates. MMRS contained the following: bacteriological peptone (Oxoid, Basingstoke, UK) 10 g, yeast extract (Merck, Darmstadt, Germany) 10 g, Tween® 80 (SigmaAldrich, St. Louis, MO, USA) 1 g, ammonium citrate 2 g, CH_3_COONa 5 g, MgSO_4_ 0.1 g, MnSO_4_ 0.05 g, Na_2_HPO_4_ 2 g (all products of SigmaAldrich) per 1 L of the medium. The pH of the media was adjusted to 6.4 and sterilised by autoclaving at 121°C for 15 min.

### Carbohydrate fermentation

Initial screening of carbohydrate fermentation was performed using the commercial API50® kit (Biomerieux, Basingstoke, UK) following the manufacturer’s instructions. Additionally, growth measurements in the presence of twelve selected carbohydrates (D-tagatose, L-sorbose, *myo-*inositol, D-lactose, D-saccharose, D-maltose, D-lyxose, pullulan, starch (all products of SigmaAldrich), amygdaline, inulin, L-arabitol (all products of AlphaAesar, Ward Hill, MA, USA) for each of the strains were performed by monitoring OD_600nm_ using a Synergy HT plate reader (BioTek Instruments, Winsooski, VT, USA). Carbohydrate solutions were prepared by the addition of the carbohydrate of interest (1 % w/v) to the MMRS followed by filter sterilisation (0.45 μm filter, Sarstedt, Wexford, Ireland). 500 μL of supplemented MMRS was inoculated with 1 % (v/v) of a bacterial culture grown in MRS at 30°C. The inoculated samples were grown at 30°C and OD_600nm_ readings were taken after 48 h, by placing 200 μL of a culture in 96 well plate. Each assay was performed in triplicate for each of the strains. Significance of differences in growth was tested by One-way Analysis of Variance (ANOVA), followed by Least Significant Test (LSD), performed in R statistical software (https://www.r-project.org/).

### Growth in the presence of heavy metal salts

Insensitivity to cadmium and arsenic ions was determined by measuring OD_600nm_ in a 96-well microplate. MRS was supplemented with increasing concentrations of CdCl_2_, and Na_2_HAsO_4_ (all products of SigmaAldrich) from 0.25 – 6 mM and autoclaved at 121°C for 15 min. Following inoculation at 1 % (v/v) with cultures grown at 30°C in the absence of heavy metal salts, growth was determined in triplicate for each concentration of heavy metal salt after 24 h of incubation at 30°C. Significance of differences in growth was tested by One-way Analysis of Variance (ANOVA), followed by Least Significant Test (LSD), performed in R statistical software.

### Determination of antibiotic resistance profiles

To determine the strains’ resistance to antibiotics, minimal inhibitory concentrations (MIC) for various antibiotics were assessed. The commercial 96-well VetMIC (SVA, Uppsala, Sweden) plates impregnated with increasing concentrations of antibiotics (gentamycin, kanamycin, streptomycin, neomycin, tetracycline, erythromycin, clindamycin, chloramphenicol, ampicillin, penicillin, vancomycin, dalfopristin, linezolid, trimethoprim, ciprofloxacin and rifampicin) were used, according to the manufacturer instructions. The growth of strains in the presence of each antibiotic was assessed after 48 h incubation at 30 °C. For each of the antibiotics, the MIC was determined as the lowest concentration of an antibiotic which prevented growth of strains. EFSA guidelines [20] were used as a reference for cut-off values for ampicillin, vancomycin, gentamicin, kanamycin, streptomycin, erythromycin, clindamycin, tetracycline and chloramphenicol resistance.

### Putrescine production

To determine if the strains produce putrescine, strains were grown in Moeller Decarboxylase broth [21]. Briefly, the broth contained bacteriological peptone (Oxoid) 5 g, meat extract (Merck) 5 g, glucose 0.5 g, bromcresol purple 0.01 g, cresol red 0.005 g, pyridoxal-5’-phosphate 0.005 g (SigmaAldrich), and L-arginine 10 g (SigmaAldrich) per 1 L of medium. The final pH was set to 6.0±0.2, and the medium was autoclaved at 121°C for 15 min. The strains were inoculated in the medium at 1 % (v/v) and incubated at 30°C for 24 h. A yellow colour indicated a negative reaction, and a purple colour indicated a positive reaction (i.e. putrescine production).

### Exopolysaccharide (EPS) production

EPS production was determined by plating strains on reconstituted MRS plates. The specific agar contained the following: bacteriological peptone (Oxoid) 10 g, yeast extract (Merck) 10 g, meat extract (Merck) 10 g, Tween®80 (SigmaAldrich) 1 g, ammonium citrate 2 g, CH_3_COONa 5 g, MgSO_4_ 0.1 g, MnSO_4_ 0.05 g, Na_2_HPO_4_ 2 g (all products of SigmaAldrich), agar (Oxoid) 15 g, and glucose or saccharose (SigmaAldrich), 20 g per 1 L of medium. Strains were inoculated on the prepared agar plates, and incubated for 48 hours at 30°C. EPS production was tested by examination of colonies for a ropy phenotype. Additionally, EPS production was determined on ruthenium agar plates, prepared as previously described [22, 23]. White colonies represent EPS-producing strains. In both assays, strain DPC1116, previously confirmed to be an EPS producer, was used as a positive control.

## Results and discussion

### Genome characteristics of DPC2071, DPC4206 and DPC4536

The three strains examined in this study were isolated as part of the non-starter microbiota of different Cheddar cheeses produced in Ireland. Strain DPC2071 was isolated from 8 week old Cheddar cheese in 1988. Strains DPC4206 and DPC4536 that share the same PFGE pattern, were isolated on separate occasions (summer and autumn 1995) from cheeses manufactured in different factories in Ireland. The fact that they were isolated within time span of several months and from different factories suggest that the same ancestral strain was used as adjunct culture during cheese manufacture, or perhaps the milk used in cheese production originated from the same producer with the ancestral strain persisting as part of milk non-starter flora.

Each of the three strains that were the subject of this study were previously designated as *Lactobacillus paracasei*, according to the results of sequencing of 16S rRNA PCR amplicons [12] and current taxonomic guidelines [24]. Further on, whole genome sequencing and assembly additionally confirmed the species. The main features of their genomes are reported in Table 1. All three genomes had a GC content of 46.3 % and genome size of approximately 3 Mbp, typically observed in *L. paracasei*.

**Table 1:** General characteristics of genomes of three strains of *Lactobacillus paracasei* [14]

	DPC2071	DPC4206	DPC4536
Genome length (bp)	2.936.872	3.095.268	3.078.575
Contigs	41	49	35
GC content	46.3 %	46.3 %	46.3%
No of CDS	2827	2951	2931
No of plasmids	11	1	0
Locus tag	BLL69	BWK52	B4Q23
Nucleotide sequence blast (BLASTn) between strains (% of query coverage, % of identity, E-value)			
	DPC2071 subject	DPC4206 subject	DPC4536 subject
DPC2071 query	-	89%, 99%, 0.0	89%, 99%, 0.0
DPC4206 query	86%, 99%, 0.0	-	99%, 99%, 0.0
DPC4536 query	86%, 99%, 0.0	99%, 99%, 0.0	-

**Fig. 1:**
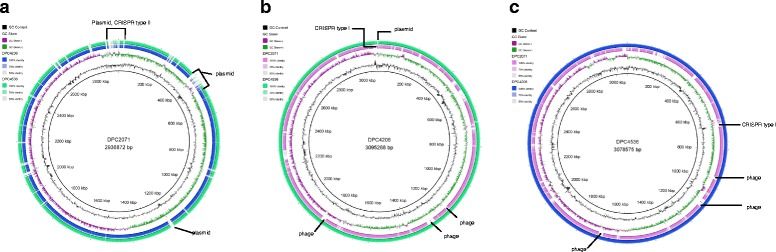
Circular maps of *Lactobacillus paracasei* strains. Maps were obtained by using (**a**) strain DPC2071, (**b**) strain DPC4206 and (**c**) strain DPC4536 as reference genomes, denoted as the inner rings. The outer rings denote genomes DPC2071 (pink) (**b**, **c**), DPC4206 (blue) (**a**, **c**) , and DPC4536 (green) (**a**, **b**)

**Fig. 2:**
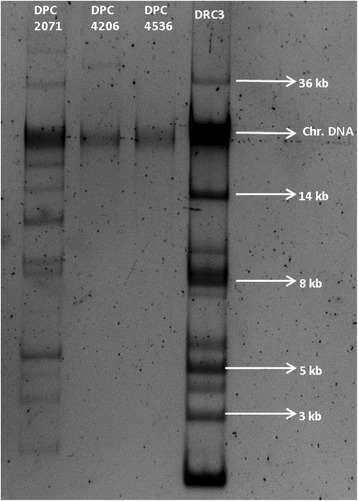
Plasmid profiles of three *Lactobacillus paracasei* strains. The plasmid profile of *Lactococcus lactis* DRC3 provides reference for estimation of plasmid sizes

In pairwise comparisons of the genomes using the Mauve alignment tool, genes specific for each of the strains were identified. In Fig. 1a, regions specific for strain DPC2071 correspond mainly to plasmids present in the strain, and a type II CRISPR system, while specific regions in DPC4206 and DPC4536 code for phage remnants and a type I CRISPR system (Fig. 1b and c). When the genomes of DPC4206 and DPC4536, strains with the same PFGE fingerprint, were aligned by BLASTn, it was shown, as expected, that the level of identity was very high (99 %, Table 1). However, the genome of DPC4206 is slightly larger, and, unlike DPC4536, it carries a single plasmid (Figs. 1b and 2). Although they shared the majority of their content, specific genes not present in the other strain were detected in both of the genomes.

### Plasmid-encoded markers suggest a more complex evolutionary route for DPC2071

Plasmids often encode genes of technological importance, such as lactose utilisation, bacteriocin production and phage resistance [25]. However, only a fraction of coding sequences (CDS) detected on plasmids of *L. paracasei* encode proteins with known function, while the remaining genes could only be annotated as encoding hypothetical proteins, with potential but still unknown function [7]. Previous reports have cited that, in general, strains of *L. paracasei* harbour up to four (strain NFBC338 [26]) or perhaps even six plasmids (strain Lpp120 [7]). However, the plasmid profile of DPC2071 suggests that this strain possesses up to 11 plasmids, although there is a possibility that some of the bands observed on the gel are due to multiple forms of the same plasmids (supercoiled, open circular or linear) (Fig. 2). The high number of plasmids was confirmed upon genome analysis, with 11 plasmid determinants (such as plasmid replication or plasmid mobilisation genes) found in different plasmid annotated contigs. Many of the predicted proteins identified on these contigs were designated as hypothetical, but certain proteins with assigned functions, such as pulullanase, thiol disulfide isomerase, collagen adhesion protein, cation transporting ATPase and pyridine-nucleotide disulfide oxidoreductase, were also identified. Apart from similarity to plasmids of *L. paracasei*, many of the plasmid-associated contigs displayed homology to plasmids of closely-related *L. rhamnosus* (Contig 38), to plasmids of the dairy species *L. helveticus* (Contig 14), of *L. plantarum* (Contig 30) or of more distantly related lactobacilli, such as *Lactobacillus hokkaidoensis* (Contig 13) and *Lactobacillus backii* (Contig 34). *L. hokkaidoensis* is a psychrophilic obligate heterofermentative LAB isolated from plant material or silage [27], while *L. backii* has been isolated from spoiled beer [28, 29]. Additionally, Contig 14 (plasmid) was abundant in genes encoding hypothetical proteins with close homologs in other genera, such as *Pediococcus*, or other unrelated lactobacilli (*L. diolivorans, L. parakefiri, L. brevis, L. suebicus*). Again, some of these species are directly connected to fermenting plant material, such as *L. suebicus* isolated from cider [30] and *L. diolivorans* isolated from spoiled cider juice [31] or maize silage [32]. Similarly, Contig 26 (not a plasmid contig) was shown to encode a large number of proteins with low level of query covers and low levels of identity with other known proteins (50 %). These proteins have been shown to be mainly involved in EPS synthesis and corresponded to proteins from other lactobacilli (*L. plantarum, L. crispatus, L. rhamnosus*) or *Oenococcus oeni*. Such a high number of plasmids and an unusual genetic content of heterogenous origin in specific genome regions points to potential interactions of DPC2071 with varying environments and the organisms therein during the evolution of this strain. It is plausible that this strain changed environments and took part in numerous genetic exchange events, which contributed to its heterogeneous gene content.

### Specificities of carbohydrate utilisation of three cheese isolates

It is believed that *Lactobacillus* species that are cheese specialists have lost numerous genes involved with carbohydrate utilisation and transcriptional regulation of carbohydrate utilisation, as the dairy niche has a very limited spectrum of available carbohydrates with lactose predominating [7]. The most restrictive sugar utilisation profiles were detected among cheese isolates, compared to plant and human isolates, which were able to use a greater variety of sugars that are available in the constantly changing habitat of these isolates [9]. Moreover, sugar utilisation profiles and gene content can indirectly indicate an organism’s previous habitats or potential interaction with strains from different ecological niches.

#### Diverse carbohydrate utilisation profiles

In order to determine sugar utilisation profiles, two approaches were used: an initial screening with the API50® kit and followed by monitoring of growth in presence of twelve selected sugars. In the API50® assay, it was shown that strains differed in the utilisation of certain sugars. Growth of strains in MMRS without added sugar did not exceed an OD_600nm_ of 0.5 after 48 h of incubation at 30°C, and this value was taken as a reference value for minimal growth of the strains.

**Fig. 3:**
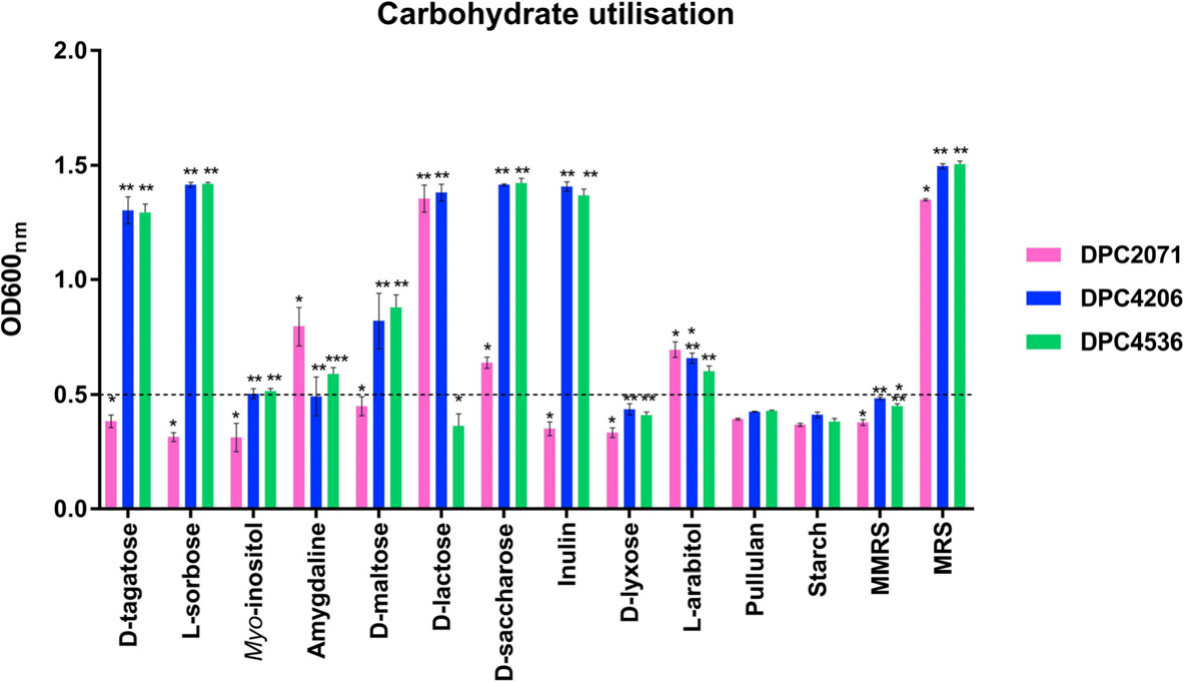
Carbohydrate utilisation. Growth of three strains of *Lactobacillus paracasei* in modified MRS (MMRS) supplemented with a single sugar in concentration 1 % (w/v) and incubated over 48 h at 30°C. Bars represent OD_600nm_ at the end of 48 h. Bars for the same sugar sharing the same asterix symbol show no statistical difference in growth (*P* > 0.05), after mean comparison by performing One-way Analysis of Variance (ANOVA) followed by Least Significant Test (LSD). The dashed line presents limit of OD_600nm_=0.5 which was used as a lower limit of growth in the presence of a carbohydrate. Legend: DPC2071 DPC4206 DPC4536

Strain DPC2071 showed limited range of carbohydrates utilised as energy sources, but used amygdaline, a plant glucoside, and grew better in the presence of L-arabitol, compared to the two other strains (colour change was more apparent). These results were confirmed in the subsequent analysis of growth in the presence of the selected sugars. Strain DPC2071 showed OD_600nm_ of 0.5 or less in the presence of D-tagatose, L-sorbose, *myo*-inositol, D-maltose and inulin and growth of OD_600nm_ = 0.64 in the presence of D-saccharose (Fig. 3). Indeed, genome comparison indicated that all genes for sorbose utilisation (L-sorbose-phosphate-reductase, transcriptional regulator, sorbitol-6-phosphate dehydrogenase, four components of sorbose specific PTS system and fructose-bisphosphate aldolase [33]) were missing in DPC2071, but were present in DPC4206 and DPC4536. Additionally, the gene encoding the first enzyme in maltose degradation, maltose phosphorylase, is interrupted by a stop codon in DPC2071, resulting in an inability to use maltose.

The two strains that shared the same genomic structure fingerprints (DPC4206 and DPC4536) showed a broader range of potential carbohydrates as energy sources and they grew in the presence of L-sorbose, D-maltose, inulin, D-tagatose and 5-ketogluconate, while in cases of *myo*-inositol and D-lyxose change of colour in API assay was small. The presence of the *fos* operon involved in utilisation of fructo-oligosaccharides, such as inulin, and the transport of free fructose [34], was confirmed in DPC4206 and DPC4536 (BWK52_0545 to BWK52_0551 in DPC4206 and B4Q23_187 to B4Q23_0193 in DPC4536) explaining the enhanced utilisation of this sugar by these two strains, compared to DPC2071, which did not possess the above mentioned genes. However, the most interesting finding of this comparison was the absence of growth of DPC4536 in the presence of lactose. The OD_600nm_ of this strain growing with lactose did not exceed 0.5, while two others reached level of 1.4 (Fig. 3). The presence of the *lacG* gene, coding for 6-phospho-beta-galactosidase (EC 3.2.1.85), the first enzyme in lactose degradation in *Lactobacillus casei* [35] in strain DPC4206 was confirmed by PCR (primers designed in this study, data not shown) and this gene was located on the single plasmid present in DPC4206 (Contig 17), which is consistent with the previous findings that lactose metabolism genes are often plasmid encoded [36]. On the other hand, both genome analysis and PCR with *lacG* specific primers showed the absence of this gene in strain DPC4536. Alternatively, in some lactobacilli (*L. helveticus* and *L. acidophilus*), lactose is firstly transported into the cell *via* lactose permease (LacS) and further metabolised by activity of beta-galactosidase, but this pathway has not been described in *L. paracasei* strains [37], and no lactose permease was identified in the genome of DPC4536.

The beta-glucoside type operons (*bgl* operons) are induced by sugars, and they are regulated by two operon products: BgIG - a transcriptional regulator (antiterminator), and BgIF - a phosphotranferase that regulates phosphorylation of BgIG and enables formation of dimers, the only active form of BgIG [38]. Five genes designated as coding for BglG transcriptional regulators (antiterminators) have been detected in the genomes of DPC4206 and DPC4536, and were not found in DPC2071. The higher number of BgIG transcriptional regulators could be connected with the broader span of sugar utilisation genes and higher number of sugars used as energy sources by these two strains compared to DPC2071, but only deeper analysis of substrate specificities of these antiterminators could reveal their actual significance in observed phenotypes.

#### The presence of specific genes for the fermentation of plant derived carbohydrates did not secure utilisation of the sugars as energy sources

*Myo*-inositol (MI) is a sugar alcohol present in soil, and it is part of phytic acid, a phosphate storage molecule in plants. It can also be metabolised by bacteria that live in soil, but it is not frequently used as an energy source in LAB [39]. So far, strains of *L. casei* are the only members of LAB that are capable of weakly metabolizing MI, but the presence of a MI metabolism cluster of genes is not a common feature of *L. casei* strains, and it does not necessarily mean that the strain carrying the cluster will use it as an energy source [40]. Previously, the presence of the complete MI utilisation operon was confirmed in the probiotic strain *L. casei* BL23 [39]. Here, strains DPC4206 and DPC4536 (BWK52_0229c to BWK52_0239 in DPC4206 and B4Q23_0140c to B4Q23_0150 in DPC4536), that possess the whole cluster of genes present in strain BL23 (level of identity was 100 %) needed for utilisation of MI did not show statistically significant growth in the presence of MI (OD_600nm_~0.55) when compared in the same media without MI added (OD_600nm_=0.5) (Fig. 3), analogous to the results from the API assay, where only a slight change of colour was observed. Similar findings, where the cluster was present but phenotype was absent, were shown for strain *L. casei* 12A [41].

Pullulan is one of the polysaccharides produced from starch, present in plant material or fermented products of plant origin. Among lactobacilli, species that are connected with plant niches (*L. amylovorus*, *L. acidophilus*, *L. amylophilus* or *L. cellobiousus)* have the ability to metabolise starch [42]. However, in dairy-related lactobacilli starch metabolism genes are not expressed due to mutation in promotor, amylase catalytic domain or signal peptide [42].

**Fig. 4:**
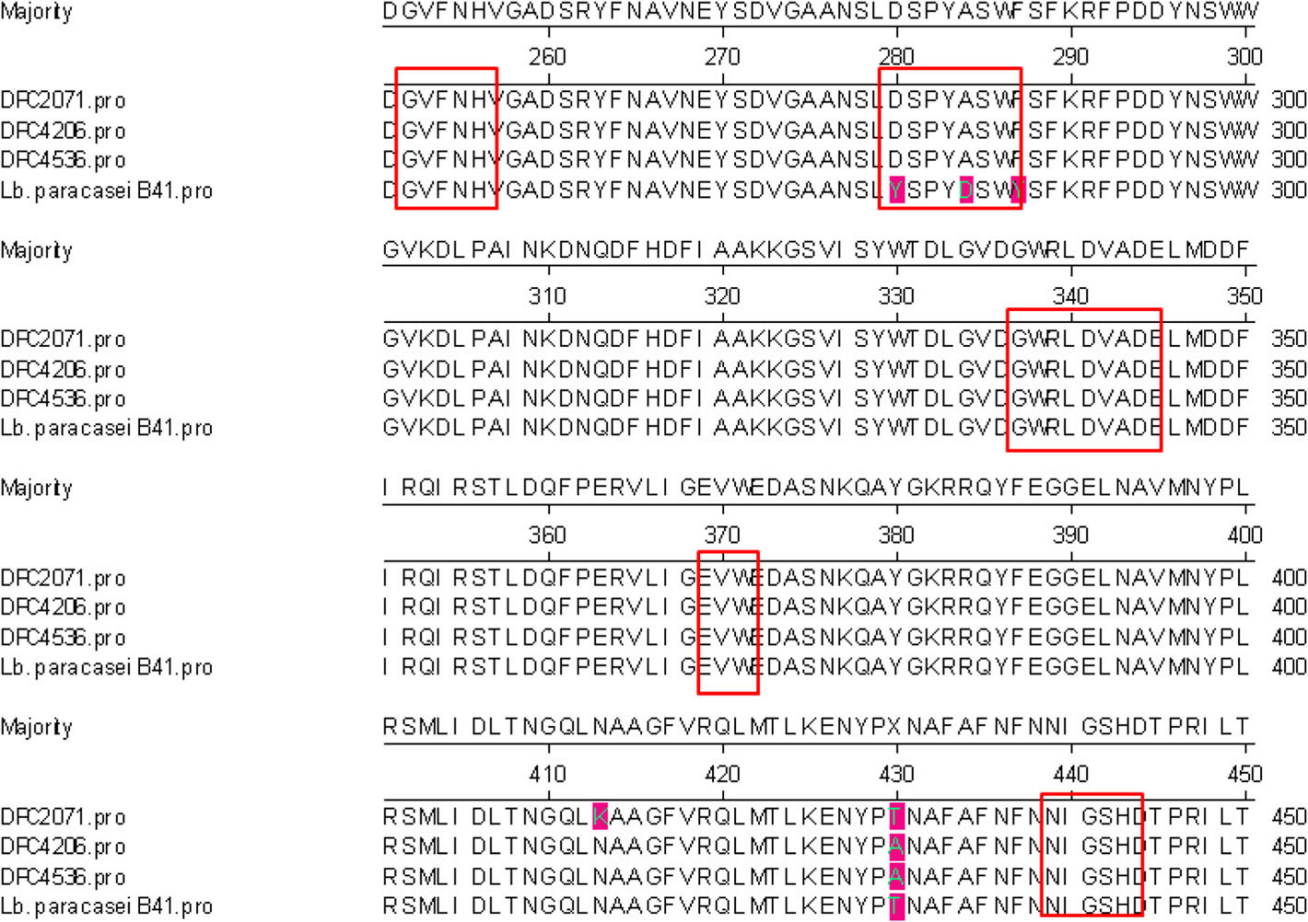
The partial representation of the alignment of amylopullulanase protein in *L. paracasei* strains. The alignment of amylopullulanase protein in strains DPC2071, DPC4206, DPC4536 and B41 was obtained by ClustalW. The conserved regions [43, 68] are boxed. The amino acids that differ from the consensus sequence are marked with the different colour

Interestingly, the genomes of all three strains analysed in this study possessed genes encoding starch degradation enzymes. Apart from neopullulanase (BLL69_0750, BWK52_1091, B4Q23_0861), and amylopullulanase (BLL69_2007c, BWK52_2351c, B4Q23_1259) encoding genes detected in all three genomes, strain DPC2071 possessed also a pullulanase encoding gene (BLL69_ 0389) located on a plasmid. However, none of the strains examined in this study could use pullulan or starch as an energy source (Fig. 3). An alignment of the amylopullulanase protein sequence from the three strains matched with the protein previously reported in *L. paracasei* B41 [43] (Fig. 4.) but the substitution of three amino acids in the catalytic domain could be the reason for the lack of the starch degrading phenotype.

### Genomic content an indicator of the flavour development potential of the cheese isolates

Flavour development in bacterial ripened cheeses originates mainly from the metabolic activities of bacteria present during ripening [44]. Although glycolysis and lipolysis contribute to the development of flavour compounds, proteolysis and amino acid metabolism particularly are seen as major contributors [45]. In the previous work of our group, it was shown that the three strains analysed in this study possess different activities of enzymes of the proteolytic cascade (cell envelope proteinase, aminopeptidases, aminotransferases) and they had variable volatile profiles in two cheese model systems [12, 13]. However, the genomic comparison of the three strains did not reveal any genetic content differences in regard to the components of proteolytic cascade, except for the methionine metabolic pathway described below. This means that the varying abilities of these strains for the development of flavour compounds most probably come as the consequence of different activities of the key enzymes or their regulation, such as the impact of coenzymes, and not due to the different number of key enzyme encoding homologs.

#### Higher number of cystathionine lyase encoding genes explains higher potential for volatile flavour compounds production in DPC4206

Volatile sulfur compounds (VSC) that arise during the microbial metabolism of sulfur compounds (methionine, cysteine) are essential for the aroma of many food products including cheese [46]. Compounds such as methanethiol, dimethyl-disulfide, dimethyl-trisulfide, dimethyl-tetrasulfide, and methional contribute to notes of onion, garlic, and cabbage in some types of cheese, such as Cheddar [47]. In bacterial amino acid metabolism, transamination represents the main pathway of amino acid degradation. The aminotransferase converts methionine to 4-methylthio-2-oxobutanoic acid, which is further converted to various VSC [46]. Besides the aminotransferase pathway, the possible involvement of cystathionine lyases in VSC production has been recently reported, although these enzymes are primarily involved in methionine biosynthesis [48]. Cystathionine lyases (cystathionine beta lyase (CBL), EC 4.4.1.8; and cystathionine gamma lyase (CGL), EC 4.4.1.1)) can use various sulfur containing substrates, including methionine, to produce methanethiol [49]. In addition, it was shown that VSC producing abilities of LAB (*Lactococcus lactis, Lactobacillus spp., Streptococcus thermophilus* and *Brevibacterium linens*) correlated with the cystathionine lyase activities [50]. Similarly, strains possessing cystathionine lyase genes used in cheese manufacture contributed to significantly higher levels of VSC at the end of ripening [51]. The overexpression of CBL in *L. helveticus* resulted in higher production of VCS from methionine and cystathionine [52].

The three genomes analysed in this study differed in content of CBL and CGL. Strain DPC2071 had one gene encoding CBL (BLL69_0664), and two genes encoding CGL (BLL69_0264, BLL69_0493c). In strain DPC4206, two CBL genes (BWK52_1002, BWK52_3061c) and two CGL genes (BWK52_0733c, BWK52_3092) were identified, while in strain DPC4536 two CBL genes (B4Q23_0772, B4Q23_2254c), and only one CGL gene (B4Q23_0463c) were present. Additionally, genes encoding cystathionine beta synthase (CBS, EC 4.2.1.22), involved in conversion of the sulfur compound homocysteine to cystathionine were present in each of the three analysed genomes. Strains DPC2071 and DPC4536 have one homolog of CBS (BLL69_0263 and B4Q23_0715, respectively), while DPC4206 has two homologs (BWK52_0941, BWK52_3091). Closer investigation showed that BWK52_3092 and BWK52_3091 in DPC4206 are located on plasmid-associated contigs, and appear to have been lost from strain DPC4536. The presence of the higher number of homologs for both CBL and CGL in strain DPC4206 could be the reason for more efficient methionine degradation observed when these strains were grown in media with an increased concentration of methionine [12]. This feature is seen as a very important attribute in cheese manufacture, and strains with optimal VCS production are potential candidates for adjunct selection.

### Resistance to heavy metals and antibiotics

**Fig. 5:**
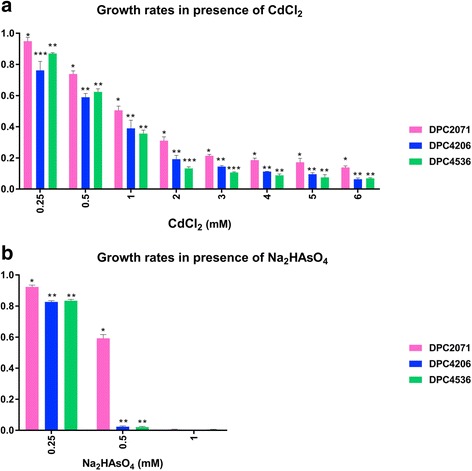
Heavy metal resistance. Growth of three *Lactobacillus paracasei* strains in MRS supplemented with corresponding heavy metal salt. Strains were inoculated (1 % (w/v)) in MRS supplemented with (**a**) CdCl_2_ and (**b**) Na_2_AsO_4_, and incubated over 48 h at 30°C. Bars represent rates between growth in the presence and absence of heavy metal salt. Bars for the same heavy metal salt concentration sharing the same asterix symbol show no statistical difference in growth (P>0.05), after mean comparison by performing One-way Analysis of Variance (ANOVA) followed by Least Significant Test (LSD). Legend: DPC2071 DPC4206 DPC4536

Bacteria possess numerous mechanisms that protect them from the increased levels of heavy metal ions they potentially encounter in the environment. The presence of these ions may result in the formation of reactive oxygen species (ROS), which disrupt the normal physiology of the cell. The growth of the strains was examined in the presence of two metal salts. Cadmium and arsine are not involved in normal metabolic processes in the cell and express toxic effects [53, 54]. Cells of the analysed strains were sensitive to CdCl_2_ at concentrations higher than 1 mM (Fig. 5a). Strain DPC2071 was the only one able to grow in 0.5 mM of Na_2_HAsO_4_, while the other two strains could not grow in this concentration of arsenic salt (Fig. 5b). The exclusive presence of the arsenical pump ATPase (BLL69_0465c) and arsenical resistance operon repressor (BLL69_0466c) in DPC2071 could explain the growth of this strain in presence of up to 0.5 mM of arsenic ion. Besides that, this strain possesses additional specific genes that could help in resisting oxidative stress caused by the elevated concentrations of heavy metals [55] and maintenance of proper protein folding [56], such as a specific glutathione reductase (BLL69_0554) and thiol disulfide isomerases (BLL69_0399, BLL69_0417).

Antibiotic resistance features were detected in all three genomes, and they comprised mainly beta lactamases, efflux pumps and glycopeptide resistance proteins, however, no differences among the resistance determinants among the genomes of analysed strains were observed. In antibiotic resistance screening, no difference in values of MIC among the strains was observed, except for neomycin, where the MIC for DPC2071 was 4 μg/mL, while for DPC4206 and DPC4536 the MIC was 16 μg/mL (data not shown). Additionally, all three strains had lower cut off values for all nine antibiotics listed in EFSA guidelines [20], and they could be considered as safe for future applications.

### DPC2071 possesses an unusual arginine metabolism gene

LAB utilize biodegradation (catabolism) of amino acids in order to gain metabolic energy or as a mechanism of resistance to a low pH environment [57]. Excessive biogenic amine (BA) production is undesirable in dairy products, since their toxic effects on humans have been shown. Putrescine, a biogenic amine originating from arginine metabolism, is one of the most common BAs produced by microorganisms used in food production, such as starter cultures, but also food contaminants, such as *Pseudomonas spp*. or *Enterobacteriaceae* [58].

**Fig. 6:**
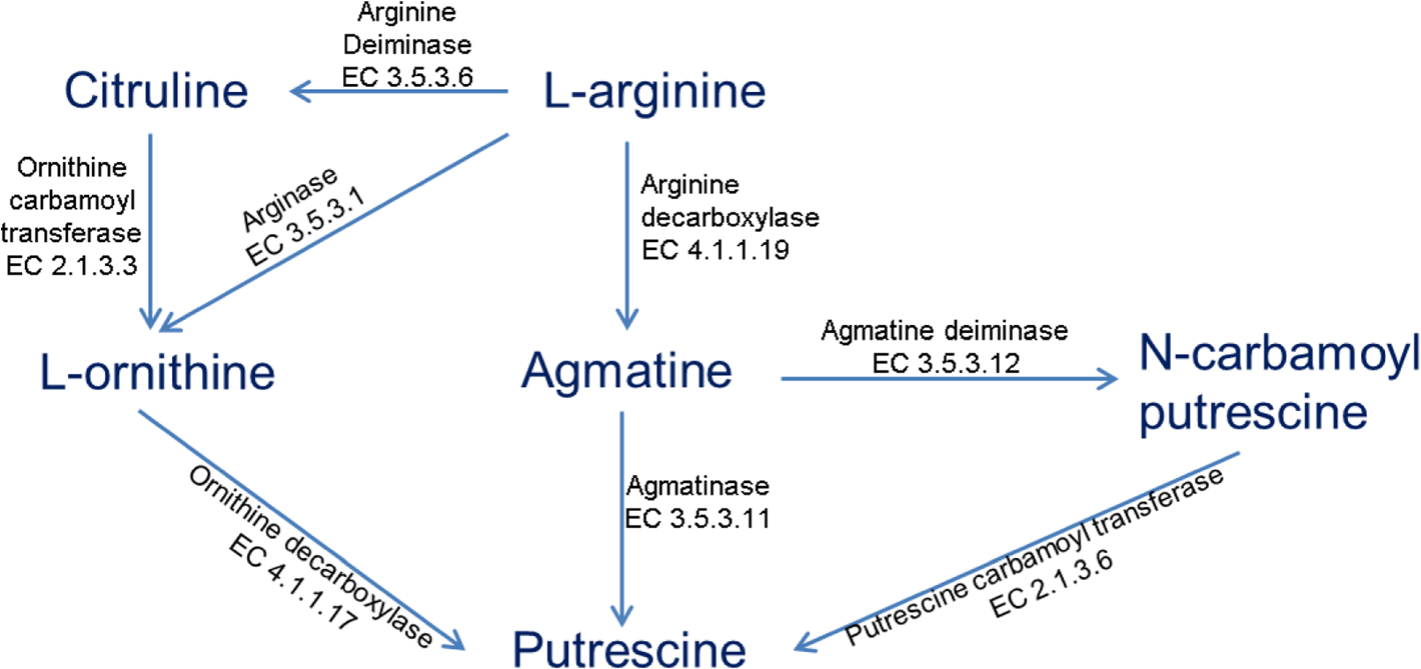
Arginine metabolic pathways in bacteria. The biogenic amines can be produced in food by microorganisms via presented metabolic pathways. Modified from [60, 61]

Generally, in Gram-positive bacteria, there are two metabolic pathways of putrescine biosynthesis: the ornithine decarboxylase pathway (ODC) and the agmatine deiminase (AgDI) pathway (Fig. 6) [58]. Additionally, a biosynthetic route where agmatine is directly converted into putrescine by the action of agmatinase (EC 3.5.3.11) has been described mainly in *Enterobacteriaceae*, but also in some dairy-borne contaminants such as *Bacillus* spp. and *Pseudomonas* spp. [59] (Fig. 6). In the publicly available genomes of *Lactobacillus paracasei*, an agmatinase-encoding gene was reported in only three strains (Lpl7, Lpl14 and CNCM I-4270), all isolated from cereals [7]. Interestingly, in the genome of DPC2071 the same gene, (BLL69_2612) was detected. The presence of agmatinase in strain DPC2071 adds to the set of unusual genes present in DPC2071 genome that link this strain to a prior plant-based niche. However, this route cannot contribute to putrescine production in DPC2071, since the gene encoding arginine decarboxylase, which transforms arginine to agmatine, was not identified.

In regard to putrescine production, although some components of putrescine synthesis pathways were detected in the genomes of the strains (such as genes encoding biodegradable ornithine decarboxylase and putrescine/spermidine transporters), arginase, which converts arginine to L-ornithine, was not detected in any of the three strains, and lack of putrescine production was confirmed in the assay for each of the three strains.

### CRISPR array content provides evidence of the independent evolution of DPC4206 and DPC4536

Clustered regularly interspaced short palindromic repeats (CRISPR) systems coupled with CRISPR associated proteins, are the most recently described phage resistance system. They are composed of a *cas* operon and a CRISPR array that contains a string of DNA repeats and spacers. Spacers correspond to foreign DNA inserted between two repeats and confirm previous encounters of the strain with different phages. Several types of CRISPR systems have been reported so far (types I, II and III), which differ in mechanism of action and the target molecule [60, 61]. Novel systems (types IV, V and VI) have been recently described [62].

In DPC2071, a type II CRISPR system was detected. Upon analysis of spacers in DPC2071 in two separate CRISPR arrays, 30 and 18 spacers were identified, 17 of which were common for both of the arrays. The genome analysis showed that the *cas9* gene, a signature gene of type II systems was broken by insertion of a transposase gene. It means that, at least in the past, this CRISPR system was efficient in conferring phage resistance, as confirmed by the presence of spacers, and the transposase gene probably had been inserted in the *cas9* recently, thus impairing its activity.

Both DPC4206 and DPC4536 possessed type I CRISPR systems. The CRISPR arrays of DPC4206 and DPC4536 contained 34 and 24 spacers, respectively, 21 of which were present in the genomes of both strains. Although the genomes of these two strains are highly similar, their CRISPR systems differ in numbers and specificity of spacers, confirming their recent divergence and independent evolutions during which they encountered different phages.

### Exopolysaccharides (EPS) biosynthesis genes did not assure EPS- producing phenotype

Many LAB produce EPS that are excreted as slime (ropy form) or remain attached to the bacterial cell wall forming capsular EPS [63, 64]. However, compared to strains isolated from the plant environment or gut isolates, dairy isolates usually carry the smallest number of EPS biosynthesis genes [7]. EPS production is considered a valuable feature, as EPS improves the rheology and texture of dairy products, such as yoghurt [65]. However, the sole presence of these enzymes and molecules is not a guarantee of EPS synthesis, as these molecules are part of numerous metabolic pathways in the cell, and should be referred to as “housekeeping enzymes” [65].

A number of genes required for EPS biosynthesis were observed in all three genomes. In addition to various EPS synthesis genes (Contig 26, reported above), strain DPC2071 possesses specific gene components of an *rfb* operon (dTDP-glucose pyrophosphorylase, dTDP-4-dehydrorhamnose 3,5-epimerase, dTDP-glucose 4,6-dehydratase and dTDP-4-dehydrorhamnose reductase (BLL669_2024c to BLL669_2027c)) that enable rhamnosyl-units to be incorporated into the repeat unit of EPS [66, 67]. Strains DPC4206 and DPC4536 also possess genes for EPS backbone production (BWK52_0503 to BWK52_0515 in DPC4206 and B4Q23_1965c to B4Q23_1977c in DPC4536), different to the ones encoded in DPC2071. However, although these genes are present in all three strains, in two experiments performed to confirm EPS production, neither a ropy phenotype on reconstituted MRS plates nor white colonies on ruthenium red milk agar plates was observed for any of the analysed strains. It is possible that either regulation of these genes leads to no EPS production under the conditions of the two experiments, or these gene clusters have an alternative function in these strains, as in ATCC334 [5]. This confirms that despite the extensive knowledge of EPS gene organisation, definite mechanisms of regulation of EPS biosynthesis remain unclear [64].

## Conclusion

This study demonstrated the variability that exists between genomes of cheese isolates of *L. paracasei*. The specific genes and specific homologs of genes detected in three strains explain some of the differences observed at the phenotypic level. Strain DPC2071 was characterised by a high number of plasmids, unusual for *Lactobacillus* strains. The genetic content of DPC2071 suggested recent transfer to the dairy niche, as well as numerous interactions with other strains of lactobacilli, not usually connected with the dairy niche. Two strains with the same PFGE pattern and with highly similar genomes (DPC4206 and DPC4536) shared most genetic content, but some differences were evident, such as the absence of the plasmid in DPC4536 and its inability to utilise lactose. Additionally, differences in spacer sequences of their CRISPR arrays confirm the strains independent encounters with phages, and thus their independent evolutions. These findings suggest that strains DPC4206 and DPC4536 probably evolved from a common ancestor and the divergence occurred in a recent event, since their genome sequences show 99% of identity. This study demonstrated the substantial level of differences in the genomic characteristics of *L. paracasei* strains isolated from the same ecological niche.

## Abbreviations

BA: Biogenic amines
CRISPR: Clustered regularly interspaced short palindromic repeats
EPS: Exopolysaccharide
MI: *Myo*-inositol
MIC: Minimal inhibitory concetration
MMRS: Modified MRS
ROS: Reactive oxygen species
VSC: Volatile sulfur compounds
